# Microhomology-mediated end joining: new players join the team

**DOI:** 10.1186/s13578-017-0136-8

**Published:** 2017-01-13

**Authors:** Hailong Wang, Xingzhi Xu

**Affiliations:** 1 Beijing Key Laboratory of DNA Damage Response and College of Life Sciences, Capital Normal University, Beijing, 100048 China; 2 Shenzhen University School of Medicine, Shenzhen, 518060 Guangdong China

**Keywords:** DNA double-strand breaks (DSBs), Microhomology-mediated end joining (MMEJ), End resection, RPA, Polθ

## Abstract

DNA double-strand breaks (DSBs) are the most deleterious type of DNA damage in cells arising from endogenous and exogenous attacks on the genomic DNA. Timely and properly repair of DSBs is important for genomic integrity and survival. MMEJ is an error-prone repair mechanism for DSBs, which relies on exposed microhomologous sequence flanking broken junction to fix DSBs in a Ku- and ligase IV-independent manner. Recently, significant progress has been made in MMEJ mechanism study. In this review, we will summarize its biochemical activities of several newly identified MMEJ factors and their biological significance.

## Background

Double-strand breaks (DSBs) are potentially lethal lesions that arise from endogenous and exogenous genotoxic agents [1, 2]. Unrepaired DSBs cause chromosome breaks and translocations that are associated with developmental defects, neurodegeneration, immunodeficiency, radiosensitivity, sterility, and cancer predisposition [3–5]. Non-homologous end joining (NHEJ) and homologous recombination (HR)-mediated DSB repair are two major pathways to fix DSBs [6, 7]. HR is generally considered to be an error-free mechanism because the identical sister chromatids are used as templates to repair DSBs when cells reside at the S and G2 phases. Ku-dependent classical non-homologous end joining (C-NHEJ) is active in all phases of the cell cycle, which can be high fidelity or associate with small alterations at junction since direct end ligation is catalyzed by DNA ligase IV [8–10]. In the absence of Ku protein or in C-NHEJ-deficient cells, resection machinery will expose extensive single strand DNA (ssDNA) which allows cells to use alternative end join (A-NHEJ) or HR as repair mechanism. A subset of A-NHEJ relies on microhomologous sequences on either side of the DSB, thus is named as microhomology-mediated end joining (MMEJ) [10–12]. MMEJ is a mutagenic DSB repair mechanism, which always associates with deletions flanking the break sites and contributes to chromosome translocations and rearrangements. Recent study indicated that MMEJ is used with appreciable frequency even when HR is available [13]. It seems that MMEJ is a crucial DSB repair mechanism for HR-defective tumors [14]. These raised the possibility that MMEJ may not just is a back-up repair mechanism. The molecular mechanism of MMEJ thus draws much attention in the field. Several important MMEJ factors have been identified recently [14–17]. Here, we will discuss biochemical properties and regulatory mechanism of these pivotal factors in MMEJ repair.

## Basic mechanisms of MMEJ

**Fig. 1:**
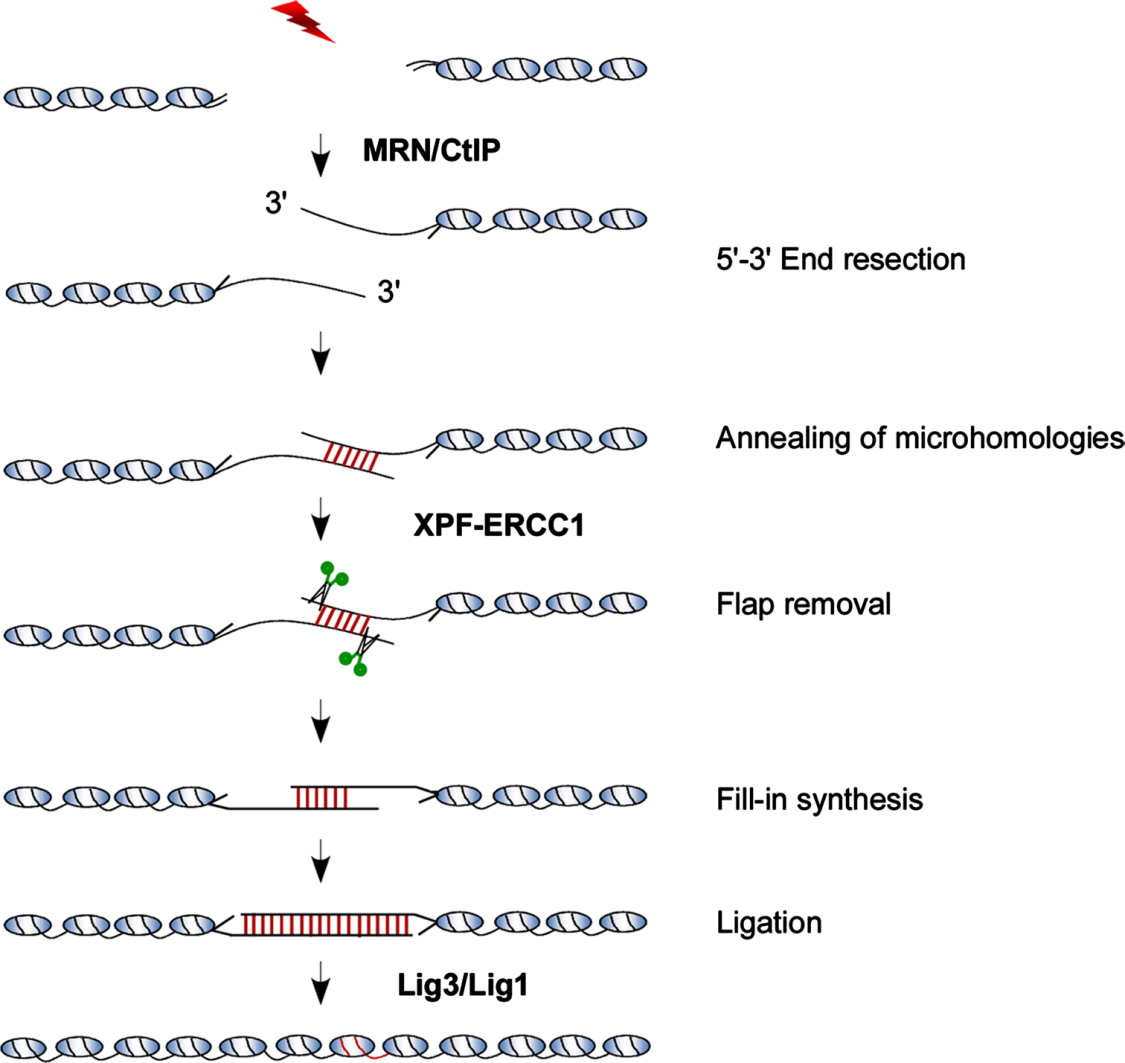
Model for MMEJ mediated DSBs repair. The first step of MMEJ is 5′–3′ end resection to expose microhomologous region, which can then anneal each other to form an intermediate with 3′-flap and gaps. The following step is flap removal and gap filling. After that, MMEJ is completed by ligation

As shown in Fig. 1, the proposed MMEJ model involves at least five steps: resection of the DSB ends, annealing of microhomologous region, removal of heterologous flaps, fill-in synthesis and ligation [17–21]. Resembling to HR-mediated DSB repair, a certain degree of end resection is also needed for MMEJ. MMEJ and HR may share the initial end resection step in DSB repair [13]. HR requires extensive end resection to recruit Rad51 recombinase and initiate homologous pairing while limited end resection is sufficient for exposing of microhomologous region and thus promoting MMEJ, Following end resection, the exposed microhomologous sequence will be annealed to form an intermediate with 3′-flap and gaps on both sides of the DSB. So far, we still do not quite clear the exactly mechanism by which microhomologous sequences move close and perform annealing. It may start with a thermodynamically-driven fashion and be regulated with some proteins factors or enzymes [16, 22]. After microhomologous annealing, the no-homologous 3′ tail (3′-heterologous flaps) must be removed to allow DNA polymerase to fill-in the gap and stabilize the annealed intermediate. Usually, this step is executed by substrate structure specific endonuclease, such as XPF/ERCC1 in mammals. The final step of MMEJ is DNA ligase III/I (Lig3/Lig1) mediated break end ligation. Obviously, after MMEJ-mediated repair, a significant part of sequence was removed from original DNA. Therefore, in nature, MMEJ is an error-prone DSB repair pathway (Fig. 1).

## Resection factors: mechanisms are still missing

In principle, both HR and MMEJ are initiated by 5′–3′ resection of DSB ends to expose ssDNA overhangs. While HR needs a long 3′-ssDNA tail to invade homologous template, MMEJ requires exposure of two microhomologous regions to anneal each other. Studies in yeast and mammalian cells indicated that DSB end resection may be carried out in two steps: Mre11 complex and Sae2/CtIP remove covalent adducts, such as bound proteins and hairpin-capped ends and initiate end resection. Sgs1/Exo1 and DNA2 in yeast or BLM (human homologue of Sgs1) and Exo1 in human cells take over to produce extended 3′-ssDNA tail [23–28]. It has been demonstrated that both Mre11 and CtIP are important for MMEJ. However, depletion of long-range resection factors including BLM/Exo1 in mammalian cells and Sgs1/Exo1 in yeast significantly increased frequency of MMEJ when the microhomologous regions close to the break site [13, 16, 29]. Possibly, down-regulation of long-range end resection may cause accumulation of short 3’tail containing DSBs which cannot be channeled to HR repair but is sufficient for exposing microhomologous region nearby DSB site and mediating MMEJ. However, we cannot rule out other possibilities yet. For example, some resection factors may harbor multiple functions. Further, the contradictory results have been obtained in studies of BRCA1, which also is a classical DSB end resection factor. BRCA1 closely associates with MRN complex and CtIP. CDK phosphorylation-mediated interaction between CtIP and BRCA1 enhances the speed of CtIP-mediated end resection [30]. Cell cycle dependent BRCA1-MRN-CtIP complex formation has been reported to play a critical role in DSB end resection and HR-mediated DSB repair in mammalian cells [31]. Early work in DT40 (chicken) B cells suggested that MMEJ is not affected by BRCA1 [32]. While, using different human cells, a recent study indicated BRCA1 may work downstream of Mre11 and CtIP to suppress MMEJ [29]. However, in MEFs cells whose telomeres were artificially uncapped, Madalena Tarsounas’s group demonstrated that CtIP and BRCA1 promote MMEJ at uncapped telomeres [33]. Obviously, more accurate systems are needed to clarify the underlining mechanism for the functional relationship between resection factors and MMEJ.

## RPA: an old soldier joined new team

**Fig. 2:**
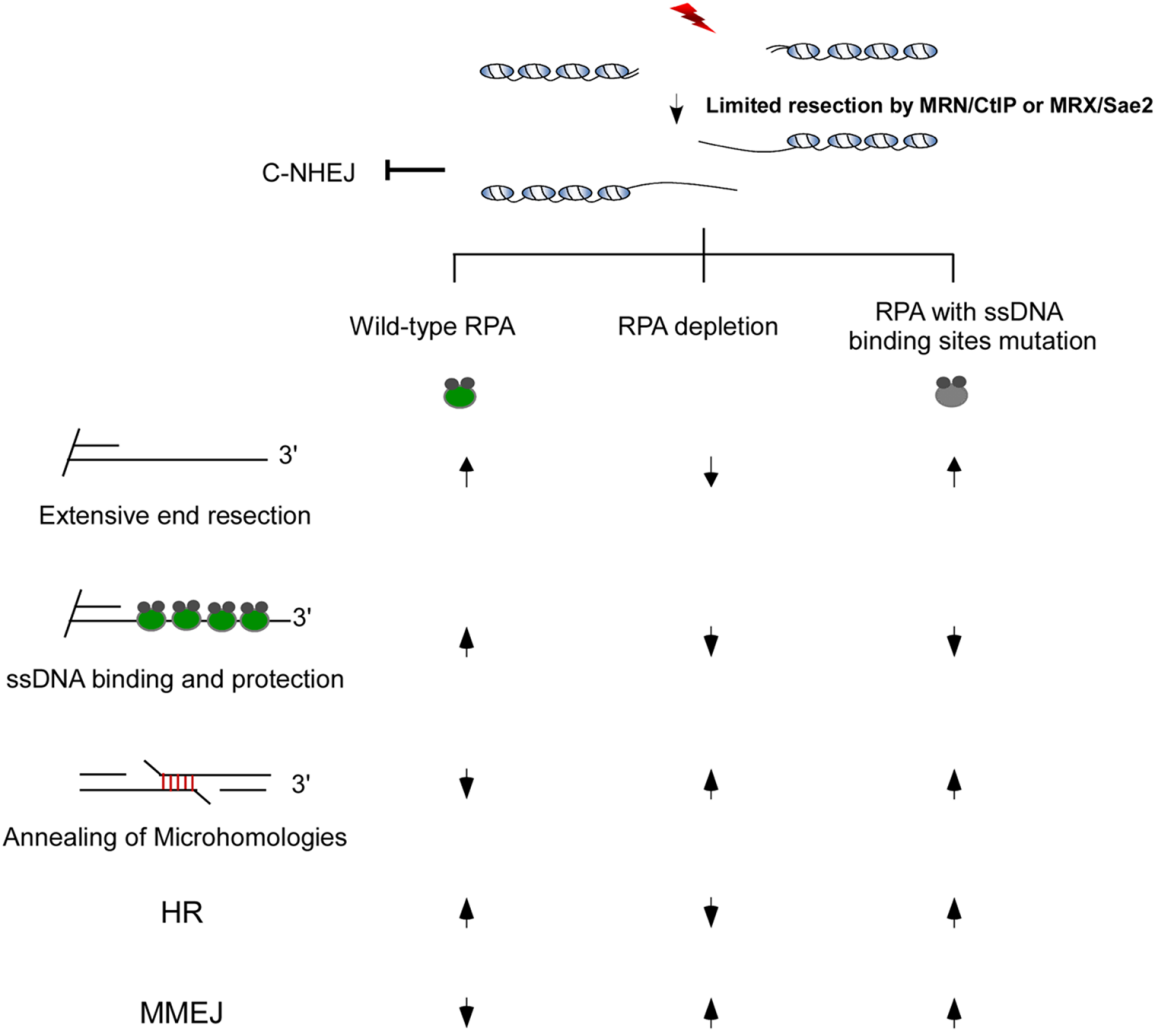
Functions of RPA and its ssDNA binding defect mutant. *Up arrow* indicating function is efficient. *Down arrow* indicating function is deficient

Replication protein A (RPA) is a conserved ssDNA binding protein in eukaryotic cells. RPA is a stable heterotrimer composed of three tightly associated subunits, namely, RPA70, RPA32, and RPA14 encoded by RFA1, RFA2 and RFA3 respectively in *Saccharomyces cerevisiae*. RPA is involved in almost all aspects of cellular DNA metabolism such as DNA replication, recombination, DNA damage checkpoints, and repair of many types of DNA damage. RPA has been reported to bind to ssDNA with much higher affinity than double strand DNA (dsDNA) or RNA. In vitro, it also harbors dsDNA helix-unwinding activity [34–38]. In budding yeast and human cells, RPA is an important component of DSB end resection machinery in previously described two-step resection model. It works together with Sgs1/DNA2 in yeast or BLM/DNA2 in human cells to promote extensive end resection [23]. After resection, RPA immediately recognizes and coats newly produced 3′-ssDNA tails by its ssDNA binding activity to stabilize ssDNA and recruit Rad51 recombinase. Rad51 recruitment was thought as a crucial step for HR repair initiation [39, 40]. Using a powerful genetic screen system, Deng et al. recently found that depletion of extensive resection factors promotes MMEJ close to the DSB site. The strains with exo1Δ sgs1Δ background show much higher proximal MMEJ frequency than wild type [16]. Actually, Lan et al. got similar results in mammalian cell using a well-designed MMEJ and HR competition repair substrate [13], indicating that long distance resection may suppress proximal MMEJ by switching repair pathway to HR. However, using hypomorphic mutant alleles of RFA1 with point mutations in the DNA-binding domain, Deng et al. showed that MMEJ can be dramatically increased without obviously decreased end resection and HR efficiency. In vitro, they also found that RPA mutants were defective in ssDNA binding and secondary structure removing. They thus concluded that RPA is a critical negative regulator of MMEJ. Independent of its end resection and HR function, ssDNA binding and dsDNA unwinding activities of RPA help it to inhibit MMEJ by preventing spontaneous annealing of microhomogous sequence flanking DSB site (Fig. 2). For the first time, Deng et al. revealed a novel function of RPA in MMEJ repair regulation [16]. Meantime, their data also help us recognize that DSB end resection may not be a rate limiting step for MMEJ although this mechanism always needs end resection to expose microhomologous region. We thus believe that other important regulatory mechanism must be existed to tightly control this error-prone repair mechanism and protect genome stability.

## Polθ: new focus

**Fig. 3:**
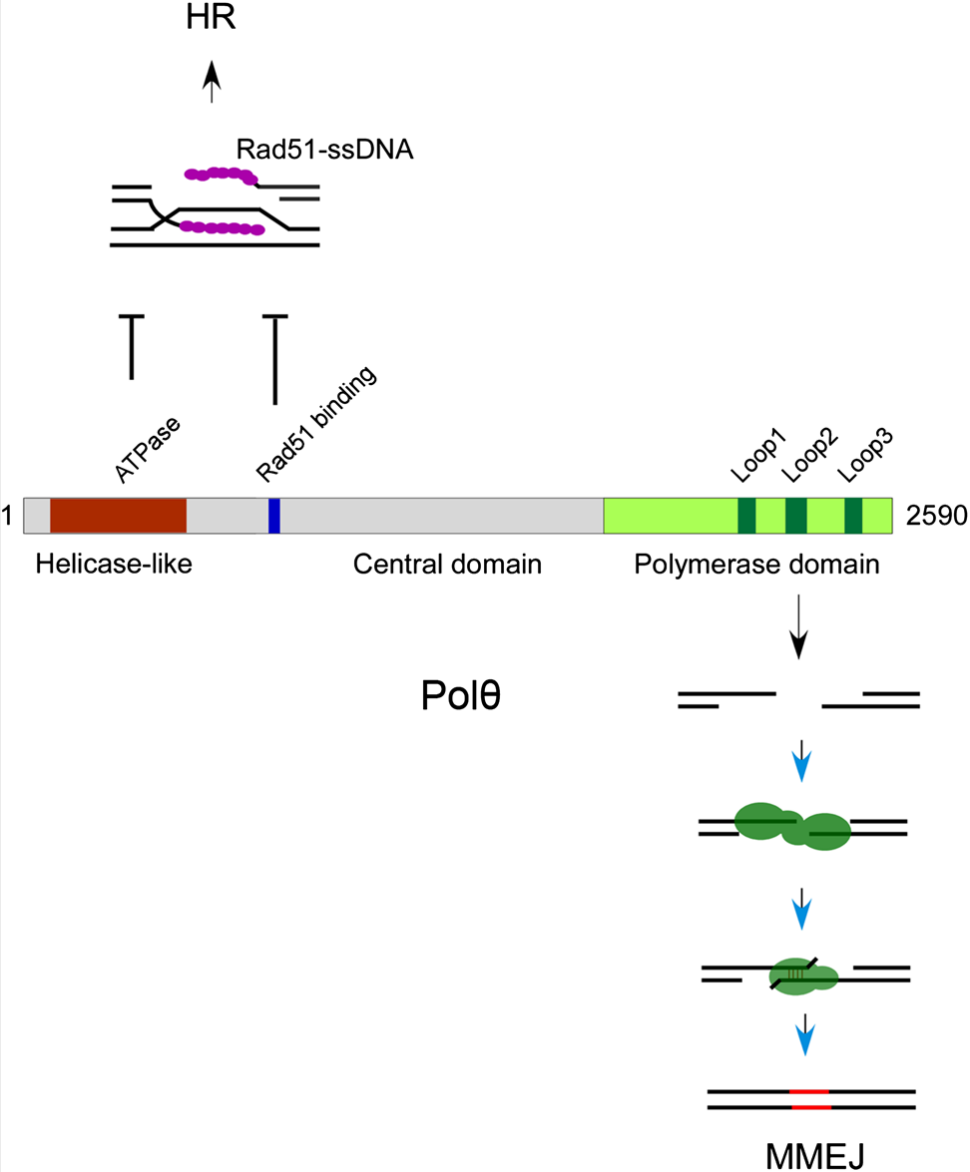
Domain structures of Polθ and its distinct functions. Polθ contain a *N*-terminal helicase like domain, a long central domian and a C-terminal polymerase domain. Polymerase domain mediates MMEJ. ATPase activity of helicase like domain and Rad51 binding motif in the central domain contribute to HR suppression

DNA polymerase theta (Polθ, also known as PolQ, encoded by *POLQ*) is a unique A-family DNA polymerase. It contains a helicase-like domain at its N terminus, which is separated from the C-terminal polymerase domain by a long, unstructured central region (Fig. 3). The helicase-like domain of Polθ is conserved among higher organisms. It shares more than 50% sequence similarity with human HELQ (also known as HEL308), which possesses dsDNA unwinding activity in vitro. However, up to now, no any strand replacement activity was identified in the helicase-like domain of Polθ either in vivo or in vitro although it showed high level of ssDNA-dependent ATPase activity [41]. The C-terminal polymerase domain of Polθ exhibits highly promiscuous enzyme activity. It exhibits low-fidelity DNA synthesis, translesion synthesis and lyase activity. Polθ also can promote extension of ssDNA and partial ssDNA substrates in an error-prone manner [42–45]. Since it was identified as the product of the *POLQ* gene more than 25 years ago, Polθ has been reported to get involved in distinct DNA damage repair pathways in different organisms. However, how its enzymatic activity link to its cellular functions still not well understood [46–50]. Recently, several reports emphasized a central role of Polθ in MMEJ-mediated DSB repair in higher organisms. Early studies in *Drosophila* indicated that Polθ promotes I-sceI-induced MMEJ, whereas polq-1 was shown to be required for MMEJ in response to replication-fork collapse at G quadruplexes in *C. elegans* [51, 52]. Recent studies in mice indicated that Polθ is associated with MMEJ-mediated fusions of dysfunctional telomeres and chromosomal translocation. Polθ was recruited to DNA damage sites induced by laser micro-irradiation in a PARP-1 dependent manner and promoted MMEJ in endonuclease-mediated reporter system [15]. Biochemical study revealed that purified human Polθ protein possesses unique MMEJ promotion activity [53]. Polθ promotes DNA synapse formation, microhomology annealing and the following synapse stabilization by catalyzing overhang extension, then stimulating MMEJ of DNA substrate containing 3′ ssDNA overhang with more than 2 bp of homology [53] The conserved insertion loop2 domain (L2 domain, Fig. 3) is important for MMEJ activity of Polθ both in vitro and in vivo. L2 domain may promote oligomerization of Polθ protein, then driving DNA end synapsis and MMEJ [53]. There are no reports to show whether the helicase-like and central domain of Polθ also directly joined in the MMEJ. However, Ceccaldi et al. identified a Rad51 binding motif in the central part of Polθ and demonstrated that ATPase activity and Rad51 binding capacity may help Polθ to block RAD51 nucleofilament assembly and HR activity, thus channelling DSB repair to MMEJ pathway. This suggests that indirect regulatory function of Polθ may also contribute to MMEJ activity.

## Concluding remarks

Increasing evidences suggest that MMEJ may not just be a back-up DSB repair mechanism. MMEJ occurs even when HR and NHEJ are intact and is essential for HR-deficient cancer cells. Therefore, it is well deserved to fully decipher the molecular mechanisms of MMEJ and its unique function in DSB repair. So far, several key factors identified in both MMEJ repair and regulation have overlapping functions with other repair pathways. Discovery of specific enzymes or protein factors that solely work in MMEJ repair pathway will help us understand the detail mechanism of MMEJ and its unique role in DSB repair and be instrumental for MMEJ-targeted drug design.
